# Enhancing the Signal of Corticomuscular Coherence

**DOI:** 10.1155/2012/451938

**Published:** 2012-05-10

**Authors:** Cristiano Micheli, Christoph Braun

**Affiliations:** ^1^Donders Institute for Brain, Cognition and Behaviour, Radboud University Nijmegen, 6525 EN Nijmegen, The Netherlands; ^2^MEG-Center, University of Tübingen, Otfried-Müller-Straße 47, 72076 Tübingen, Germany; ^3^Center for Mind/Brain Sciences (CIMeC), University of Trento, Via delle Regole 101, 38100 Trento, Italy; ^4^Department of Cognitive and Educational Sciences (DiSCoF), University of Trento, Via delle Regole 101, 38100 Trento, Italy

## Abstract

The availability of multichannel neuroimaging techniques, such as MEG and EEG, provides us with detailed topographical information of the recorded magnetic and electric signals and therefore gives us a good overview on the concomitant signals generated in the brain. To assess the location and the temporal dynamics of neuronal sources with noninvasive recordings, reconstruction tools such as beamformers have been shown to be useful. In the current study, we are in particular interested in cortical motor control involved in the isometric contraction of finger muscles. To this end we are measuring the interaction between the dynamics of brain signals and the electrical activity of hand muscles. We were interested to find out whether in addition to the well-known correlated activity between contralateral primary motor cortex and the hand muscles, additional functional connections can be demonstrated. We adopted coherence as a functional index and propose a so-called nulling beamformer method which is computationally efficient and addresses the localization of multiple correlated sources. In simulations of cortico-motor coherence, the proposed method was able to correctly localize secondary sources. The application of the approach on real electromyographic and magnetoencephalographic data collected during an isometric contraction and rest revealed an additional activity in the hemisphere ipsilateral to the hand involved in the task.

## 1. Introduction

One of the aims in applying recording techniques such as EEG and MEG, but also more recently ECoG, iEEG and laminar recordings, is to exploit the fine grained temporal resolution of the neuronal activity of the brain in order to quantify functional connectivity [1] between different brain areas and between the brain and external signals that might either be stimulation sequences or muscular activity in motor tasks.

Strong progress has been reported in the detection of brain areas revealing correlated activity by means of advanced source reconstruction techniques [2–8] such as beamformer. This methodology has successfully been applied in noninvasive EEG and MEG recordings and more recently also with invasive measurement of human brain activity [9]. Beamformers are capable of improving the signal-to-noise ratio (SNR) of the acquired brain activity [10, 11], and thus enable a better reconstruction of the functional connectivity patterns among brain regions and between brain regions and external signals, than could be obtained by sensor level analysis.

In the present study, we focus on corticomotor connectivity reflecting peripheral motor control. To this aim, we acquired MEG data and the electromyogram (EMG) of finger muscles involved in a pinch grip task requiring isometric contraction. We used the oscillatory modulation of EMG activity to identify driving brain areas. As index for the synchronization between the external EMG signal and brain areas' specific activity we have chosen corticomuscular coherence (CMC, [12–15]).

Assuming a single driving brain area beamformer performs well as long as the signal-to-noise ratio is high enough. Yet, assuming multiple brain sources whose activity is correlated with the EMG signal, beamformers might fail to localize the respective brain regions because the algorithm requires that the brain sources to be detected are linearly independent from each other [16]. However, in case of multiple sources being part of a larger network, this assumption might not be true and the beamformer approach will give faulty results.

Most importantly, if the muscular activity is modulated by the activity of more than one brain region at a time, a conventional beamformer will most likely localize only the most prominent modulatory source. It is well known from previous studies [5, 17] that the reconstruction of the brain sources correlated with an external source will result in the localization of the most correlated area and the cancellation of the other source. Given that it is not possible to localize multiple sources at a time, we identify the neural motor network in steps by progressively cancelling the most correlated sources with a technique called “nulling beamformer” [2, 6].

The present paper proposes a general pipeline which can be used to characterize cortico-motor connectivity in the source domain. We first adopt a DICS (dynamic imaging of coherent sources, [18]) beamformer and use coherence with the peripheral EMG signal as a metric to intercept the brain source showing strongest coherence. We then suppress this primary source by means of a nulling beamformer in order to uncover possible secondary (weaker) sources showing coherence with the EMG signal.

Additionally, we make use of a complementary step based on a subspace reprojection method [19, 20] to better identify both primary and secondary locations. This technique is necessary since the data is noisy and the localization can be improved by removing the local uncorrelated noise superimposed to the source signal. To demonstrate the validity of our approaches we applied them to simulated data of corticomotor coherence and to recordings in a real world experiment.

## 2. Methods

### 2.1. The DICS Beamformer with an External Reference Channel

The linearly constrained minimum norm (LCMV) beamformer is a spatial filter, which is used to separate the signal coming from a location of interest from other interferences. For a source, the time course of its activity can be expressed as the product of the raw data by the weights of the spatial filter:

(1) d(r,t)=w(r)B(t)

or, in the frequency domain:

(2) d(r,f)=w(r,f)F(f).

**B**(*t*) indicates the raw data, **F**(*f*) the Fourier transform of the data, **w**(**r**) and **w**(**r**, *f*) the weights calculated with a time [16] or frequency domain [18] LCMV beamformer, and **d**(**r**, *t*) and **d**(**r**, *f*) the time course or the Fourier transform of the reconstructed dipolar source. The calculation of the spatial filter implies finding the multiplying weights that obey certain conditions.

Technically this is achieved by imposing that the variance of the source location is minimal, with the additional linear constraint that the signal originating from that location of interest (we call it the “virtual sensor”) is retained. This is equivalent to writing

(3) $$\underset{w}{\text{argmin}}\left(w^{T}C_{\text{MEG}}w\right),\;\;wL\left(r\right)=I,$$

where **I** indicates the unitary matrix, *T* is the matrix transpose operator, **C**_MEG_ is the *N* × *N* covariance matrix of the MEG channels, and **L**(**r**) is the lead field for the location **r**. By means of Lagrange function minimization the equation above leads to the following solution [5]:

(4) $$w\left(r\right)={\left(L{\left(r\right)}^{T}\cdot {C_{\text{MEG}}}^{-1}\cdot L\left(r\right)\right)}^{-1}L{\left(r\right)}^{T}{C_{\text{MEG}}}^{-1},$$

where −1 indicates the matrix inversion.

Hence the beamformer spatial filter is characterized by its weights: a set of *N* coefficients, being *N* the number of MEG channels. As a consequence all sensor level measures such as power, cross-spectral density, and coherence are translated into virtual sensors measures by a simple multiplication with the weights. As such

(5) d(r,f)=wF(f)

is the Fourier transform of the source

(6) $$P_{\text{MEG}}\left(r\right)=w^{T}F^{T}Fw\;\text{or}\;\text{alternatively}\;P_{\text{MEG}}\left(r\right)=w^{T}C_{\text{MEG}}w$$

is the power of the source.

(7) $$\text{csd}\left(r\right)=w^{T}C_{\text{REF}}$$

is the cross-spectral density of the source, with **C**_REF_  being the cross-spectral density between the MEG channels and an external source

(8) $$\text{coh}\left(r\right)=\frac{\text{csd}{\left(r\right)}^{2}}{P_{\text{EMG}}\cdot P_{\text{MEG}}\left(r\right)}$$

is the coherence between the source and the external signal and **P**_EMG_ and **P**_MEG_(**r**), are respectively, EMG and MEG sensors' power (at frequency *f*).

This particular fashion of beamformer is called reference-channel DICS and makes use of EMG-MEG coherence to localize brain synchronicity. It is important to note that the coherence parameter can be visualized as a 3D map in the head space of the subject and that DICS beamformer is frequency specific, as such the parameters in the previous equations are defined for one frequency bin at a time. The localization of the brain source which swings in synch with the EMG is operationalized by taking the maximum of the DICS coherence 3D map.

The coherence peak in the map, localized by means of DICS, takes the name of primary source or main source. To verify the reliability of DICS localization, we perform simulations with a priori known source locations and we evaluate the significance of the coherence in subjects' data according to [15], as

(9) $$\text{Significance}=1-{\left(1-\alpha \right)}^{1/(L-1)},$$

where *α* indicates the confidence level (95% in our case or *α* = 0.95) and *L* the number of epochs.

### 2.2. The Nulling Beamformer

This technique is used to suppress the main activity, after its localization by means of the formerly described DICS technique. The implementation of the nulling beamformer begins with the construction of a modified lead field matrix $\tilde{L}$, obtained by adding a term **C**_*S*_ on the right side of the lead field matrix **L** from the previous equations. This method requires the preselection of a ROI where the activity of the source has to be suppressed and defines the matrix **C**_*S*_ as composed by the columns of the sources' lead fields to be cancelled. In formulas

(10) $$\begin{array}{c} \tilde{L}=\left[L\;C_{S}\right], \end{array}$$

(11) $$w\left(r\right)={\left(\tilde{L}{\left(r\right)}^{T}\cdot {C_{\text{MEG}}}^{-1}\cdot \tilde{L}\left(r\right)\right)}^{-1}\;\tilde{L}{\left(r\right)}^{T}{C_{\text{MEG}}}^{-1}.$$

All quantities such as coherence and source power are defined as in the previous paragraph. Subsequently, a nulling constraint is imposed on the weights such as their spatial band pass and band stop characteristics are defined. This constraint is implemented by the multiplication with a coefficient **c** so that

(12) $$w_{N}\left(r\right)=cw\left(r\right)$$

with **c** = |1  0  0 ⋯ 0| for the scalar and **c** = [I 0] for the vector beamformer, where unity values correspond to the band pass part of the filter and zeros to the band stop.

One of the drawbacks of this technique is that the degrees of freedom (defined as *M* − 3∗*J* − 1 ([21])) of the inversion term in (11) diminishes by a factor of 3∗*J* (*M* = number of channels, *J* = number of dipoles in the ROI [5]), making the aforementioned matrix close to singular. Therefore the choice of the ROI's radius is a trade-off between the extent of the area to be suppressed and the available degrees of freedom. A workaround to overcome the problem of insufficient degrees of freedom is to reduce the rank of the matrix **C**_*S*_ by means of a singular value decomposition (as proposed in [6]). We normally apply the dimensionality reduction with a rejection percentage of 1 to 10% of the smallest eigenvalues.

The regional nulling beamformer is applied in this case to get rid of the primary cortico-muscular coherent activity to be able to visualize secondary sources. The attenuation on the unwanted primary activity in fact has the net effect of enhancing sources otherwise masked by the main localization, as demonstrated in Dalal's work [6].

#### 2.2.1. Implementation Details

All analysis are run in Matlab (The Mathworks, Natick, MA, USA) and make use of FieldTrip, an open source toolbox for data analysis [22]. The nulling beamformer analysis is a novel and “nonstandard” part in the analysis pipeline (see Figure 2). Therefore its implementation is described in further details. All initial steps (referred to as “preprocessing”) are documented in the FieldTrip documentation pages: http://fieldtrip.fcdonders.nl/tutorial/beamformer. The names in italics that the reader encounters in this paragraph refer to specific FieldTrip instructions as they are typed in the Matlab environment.

The output variables of the previous analysis (see Figure 2, box “Inverse solution”) are (a) the volume conductor (describing the geometry of the head), (b) the positions of the sensors, (c) the trial-wise Fourier coefficients of the MEG channels, (d) the trial-wise Fourier coefficients of the EMG channel, (e) a “grid” structure containing (among others) the positions of the sources in 3D Cartesian coordinates and the corresponding lead fields describing the dipolar sources (forward solution), and (f) the coordinates of the point of maximal coherence as localized from DICS method. All reported quantities are stored as variables in the standard FieldTrip data structures (for a thorough reference on data structures please refer to the following page: http://fieldtrip.fcdonders.nl/faq/how_are_the_various_data_structures_defined).

Operatively the nulling beamformer involves the following steps:

1. definition of the ROI extent and construction of the  **C**_*S*_  matrix in (10);
2. modification of the leadfield matrices contained in the grid variable (the structure described previously at step (e)), by adding the columns of the  **C**_*S*_  matrix (or its reduced version) on the right side of each leadfield matrix;
3. calculation of the beamformer weights, as described in (11) (implemented by the function *ft_sourceanalysis*; the configuration options require *cfg.keepfilter =“yes”* and *cfg.method = “dics”* as input arguments for the function);
4. multiplication of the calculated weights by the matrix **c** in (12), which defines the “band-pass” (=1) and “band stop” (=0) terms of each spatial filter;
5. calculation of the EMG-MEG cross-spectral density (csd) matrix for a defined frequency, using the function *ft_frequancyanalysis* (with the option *cfg.output = “csdandpower”*); note that the MEG and EMG datasets have to be previously appended (*ft_appenddata*);
6. creation of a *filter* field added to the grid structure (the previously mentioned FieldTrip structure, see step (e)) and containing the weights calculated in step 3;
7. projection of the previously calculated cross-spectral density (point 5) through to the weights. The projection step is implemented internally in the *ft_sourceanalysis* function and is accomplished by setting the arguments *cfg.method = “dics”* and *cfg.refchan = “EMGchannelname”*.

Note that each dipole's leadfield matrix has to be normalized by its Frobenius norm so that the beamformer localization is not affected by the depth bias [23]. One important issue regarding the nulling procedure is the inversion of the ${\left(\tilde{L}(r\right)}^{T}\cdot {C_{\text{MEG}}}^{-1}\cdot \tilde{L}(r))$ term in (11), which becomes rank deficient due to the modification of the leadfields [5]. To solve the problem the Moore-Penrose pseudoinverse [24] is usually applied (implemented in the Matlab function “*pinv*”; the pseudoinverse function used in FieldTrip, which is also called “*pinv*” is a subfunction of the “*beamformer_dics.m*” private function. In this version of *pinv* the tolerance is increased by a factor of 10 with respect to the standard *pinv* function). Most importantly the number of trials has to be superior or comparable to the number of MEG channels (a VSM-CTF system has 275, see “The Experiment” in paragraph 2.6.2) in order to estimate an unbiased channel level csd matrix (**C**_MEG_  matrix in (11)).

### 2.3. The Data Subspace Reprojection

This technique has the aim of rejecting the noise in a ROI. The implementation consists in the definition of a geometrical region of interest and in the calculation of the Gram matrix related to the specific ROI, called Ω. The theoretical expression of the Gram matrix for a discrete points ROI Ω is:

(13) $$G=\overset{}{\underset{r_{i}\in \Omega }{\sum }}L\left(r_{i}\right)L^{T}\left(r_{i}\right),$$

where **L**(**r**_*i*_) is the lead field defined for the voxel position **r**_*i*_.

Operatively, the algorithm requires to left multiply the data matrix **B**(*t*) by a matrix **E**, whose columns are formed by the *S* larger eigenvectors of the Gram matrix (*S* < *N*: number of sensors). The selection criterion for the *S* is based on the variance explained by the first *S* sorted eigenvalues, according to:

(14) $$\varepsilon =\frac{\sum_{i=1}^{S}\text{eig}{\left(G\right)}_{i}}{\sum_{i=1}^{N}\text{eig}{\left(G\right)}_{i}}\times 100.$$

In formula, we can call **E**_*S*_  the matrix representing the *S* eigenvectors corresponding to the first *S* largest eigenvalues of **G**, so that:

(15) $$B_{\text{DENOISE}}\left(t\right)=E_{S}E_{S}^{T}B\left(t\right).$$

The described approach has been used in the context of EEG data analysis to get rid of spatial specific noise in a region of interest [19]. The consequence of the double matrix multiplication is equivalent to a PCA rejection of the small components and then a reprojection on the data space. This has the effect of reducing the noise due to the data outside of a certain ROI of interest and can be applied both to the main and to the secondary sources localizations in order to improve the signal to noise ratio of the local coherence.

### 2.4. The Complete Pipeline in a Flow Chart

A schematic representation of the processing which applies the proposed methods in a consistent pipeline is presented in Figure 2, where each block depicts a single step of the processing flow. In particular the forward solution block implements the necessary steps to obtain the lead field matrices, used by the beamformer algorithm [5]. We make use of the Nolte solution [25], whereas more recent implementations of the MEG forward solution are available [26].

The preprocessing can be different according to the recordings and in the case of MEG implies the rejection of artifacts as described in [27]. Notably we make also use of independent component analysis (ICA) to accomplish this task [28]. No rectification is applied to the EMG since it has been recently objected to affect negatively the quality of the CMC analysis [29]. The spectral analysis makes use of multiple tapers to calculate both the spectral power and the cross-density matrix between MEG channels and the myography as described in [30]. We process epochs of 1 second with a number of 5 tapers.

The first step coming after frequency analysis is the DICS beamformer, which takes as inputs the spectra of both MEG and EMG signals, their cross spectrum and the lead fields resulting from forward model calculation. The visualization step generates a map of cortico-muscular coherence in three orthographic projections and highlights the maximum value (main activation). The region of interest (ROI) analysis implies the selection of the sources around the peak of activity in a radius of 3 cm from it. This step results in the extraction of the lead fields corresponding to the sources included in the ROI. These lead fields are used both for the nulling beamformer and for the beamspace reprojection method.

Successively the pipeline is composed by two additional steps: one responsible for the spatial band stop filtering of the data and the subsequent localization of the secondary CMC sources, the second responsible for the visualization of the results. The subspace reprojection technique is depicted in blue in Figure 2 and is generally applied to enhance the SNR of the localized sources.

### 2.5. The Statistical Analysis

The threshold of significance for the coherence maps was determined by a randomization. The first step of the statistical analysis shuffles the trials of the EMG's Fourier transform and generates a new set of complex coefficients at a certain frequency (the peak of sensor level coherency). For the specific goal of this paper we use *N*_perm_ = 100 permutations. Successively we calculate the beamformer coherence for all iterations, which results in as many vectors as numbers of permutations.

The *P* values for the randomization test (see paragraph 2.3 of Maris and Oostenveld paper [31]) are obtained from

(16) $$P{\left(i\right)}_{k}\;=\;\frac{\Sigma_{k}\delta \left(\text{coh}{\left(i\right)}_{k}-\text{coh}\left(i\right)\right)}{N_{\text{perm}}},$$

where *i* = 1 … *N*_vox_ is the voxel index, *k* is the permutation index (*k* = 1 … *N*_perm_), coh_*k*_(*i*) is the randomized coherence for voxel *i* at permutation *k*, coh(*i*) is the nonrandomized coherence calculated at voxel *i* and *δ* is a Kronecker function, which follows the rules:

(17) $$\delta =1\;\text{if}\;{\text{coh}}_{k}-\text{coh}>0,\;\;\delta =0\;\text{if}\;{\text{coh}}_{k}-\text{coh}\leq 0.$$

The resulting *P* values which are smaller than a significance threshold for the statistical test (we choose 5%) are selected and correspond to spatial locations in the coherence map. The minimum coherence value of the extracted pool of voxels represents the empirical threshold.

### 2.6. Details of the Experiment and the Simulations

#### 2.6.1. The Simulations

The simulation paradigm consists of two datasets: (1) a simulated MEG field containing two oscillating sources which are correlated with a simulated EMG signal, (2) a simulated MEG field containing one oscillatory source and a simulated EMG signal correlated with it. The first simulation is meant to show the effectiveness of the nulling beamformer in the suppression of a brain source and in the enhancement of the secondary one. In the second simulation, noise is added gradually in 10 different datasets, to show the degradation of the beamformer signal and the partial enhancement of the SNR due to the data reprojection technique using a ROI of 2 cm around the source and a number of *ε* = 10^−7^ to select the largest eigenvalues.

**Figure 1:**
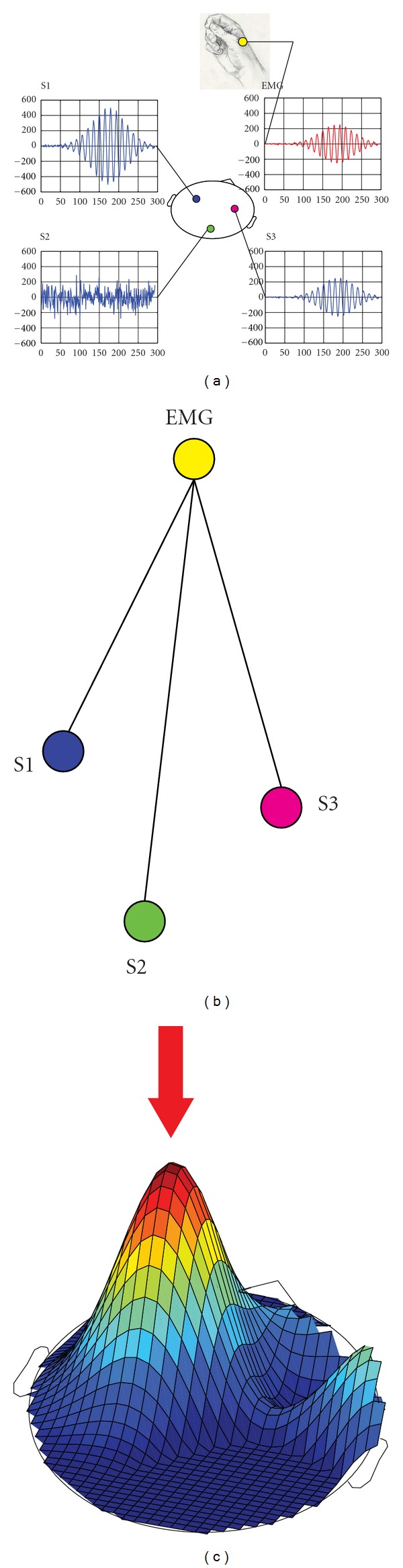
A representation of the motor network and of how the connectivity is calculated. (a) A representation of simulated brain sources in a volumetric slice (blue time courses, marked as S1, S2, and S3) and hand surface myography correlated with tracks S2 and S3 (EMG, red time course). (b) The scheme of functional connectivity. Coherence is calculated between EMG and all other sources. (c) A schematic output of DICS beamformer coherence. Note the presence of only one peak corresponding with the source having the highest SNR (S1). The red arrow indicates that this peak has to be suppressed by the nulling beamformer in order to localize the second EMG-correlated source (S3).

**Figure 2:**
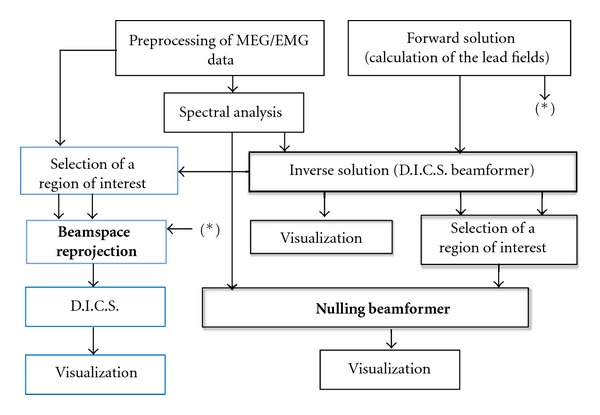
Scheme of the processing for beamformer localization of EMG coherent sources. The boxes indicate the methods described in this paragraph, with particular emphasis for the beamformer steps (shadowed boxes). The bold text in the boxes represents the algorithms contributed in the present paper. The light blue boxes refer to the beamspace reprojection pipeline, used to enhance the visualization of the main CMC source.

The time courses of both simulation 1 and simulation 2 are generated by multiplying a Gaussian envelope with a 20 Hz sinusoidal signal in an epoch of 1 second (see Figure 1). The peak of the sinusoid is nominally fixed at 0.5 seconds and two jitters are defined across the 100 trials for phase and amplitude of the sinusoid. The coupling between and external signal and the simulated sources are defined by assigning a deterministic and a random component to the aforementioned jitters, according to the formula:

(18) $$\;\begin{array}{cc} & {\text{MEG}}_{{\text{jitter}}_{i}}=\left[\det\_i*\text{CS}+\text{rand}\_i*\left(1-CS\right)\right]*\text{nominal},\\ & \text{EMG}\_\text{jitter}\_i=\det\_i*\text{nominal}, \end{array}$$

where MEG_jitter_*i* and EMG_jitter_*i* represent the jitter for EMG and MEG time courses at epoch *i*, CS is the coupling strength (a parameter defined between 0—no coupling- and 1—perfect coupling-), det_*i* is a realization of random noise which is common to EMG and MEG signals, rand_*i* is a realization of random noise that is present only in MEG signal, and *nominal* is the nominal value of the jitter parameter (phase or amplitude jitter).

The time courses are then multiplied for the lead fields of two sources defined in positions (0,−4,10) and (0,4,10) cm in head Cartesian coordinates according to the CTF axes conventions [32]. The resulting simulated magnetic field is then added with random noise with varying intensity. In the first simulation the intensity varies from 10 to 100 times the root mean square value (rms) of the clean dataset, whereas in the second simulation the level of superimposed noise is 0.2 times the rms of the clean dataset.

The rms of a dataset is defined as

(19) $$\text{rms}=\sqrt{\frac{\sum_{i}^{}{\left(x_{i}\right)}^{2}}{N}},$$

where *x*_*i*_ is a sample of the dataset and *N* is the total number of samples. The coupling strength for the first simulation is 1 for all jitters, whereas for the second simulation we choose 1 for the amplitude jitter and 0.9 for the phase jitter.

#### 2.6.2. The Experiment

The experiment was run with a whole head, 275 channels MEG system (CTF/VSM Inc. Port Coquitlam, Canada) equipped with first-order axial gradiometers with 5 cm baseline and installed inside a magnetically shielded room (MSR, Vacuumschmelze Hanau, Germany). During the recording time the subject sat on a chair whose position and height could be regulated on demand. Its position was set such that the subject could see a feedback image projected on a screen of dimensions 42 × 32 cm (width × height) situated at a distance of 0.4/0.5 m. An ad hoc built pinch device, which had a strain gauge force transducer mounted on it, was attached to the armrest of the chair in a position that was comfortably reachable by the subject's right hand.

Five subjects (all aged between 30 and 35, all right handed, 4 males and 1 female) were selected for the experiment with the aim of carrying out a subject-specific analysis (no grand averages were planned for this experiment). A muscular activity (EMG) was recorded using bipolar derivations with electrodes of 11 mm diameter (In Vivo Metric, Healdsburg USA) mounted on the dorsal surface of first dorsal interosseous muscle at 20 mm distance from each other. Electrode gel “Abralyt light” (Falk Minow Services, Herrsching, Germany) was used to establish electrical contact between skin and electrodes with impedances below 20 kOhm. The EMG was digitized and stored in the same datasets as the MEG recordings. The subject had to grasp the pinch device and elicit a continuous constant force of 1 Newton for the whole duration of the experiment.

## 3. Results

### 3.1. The Simulations' Results

**Figure 3:**
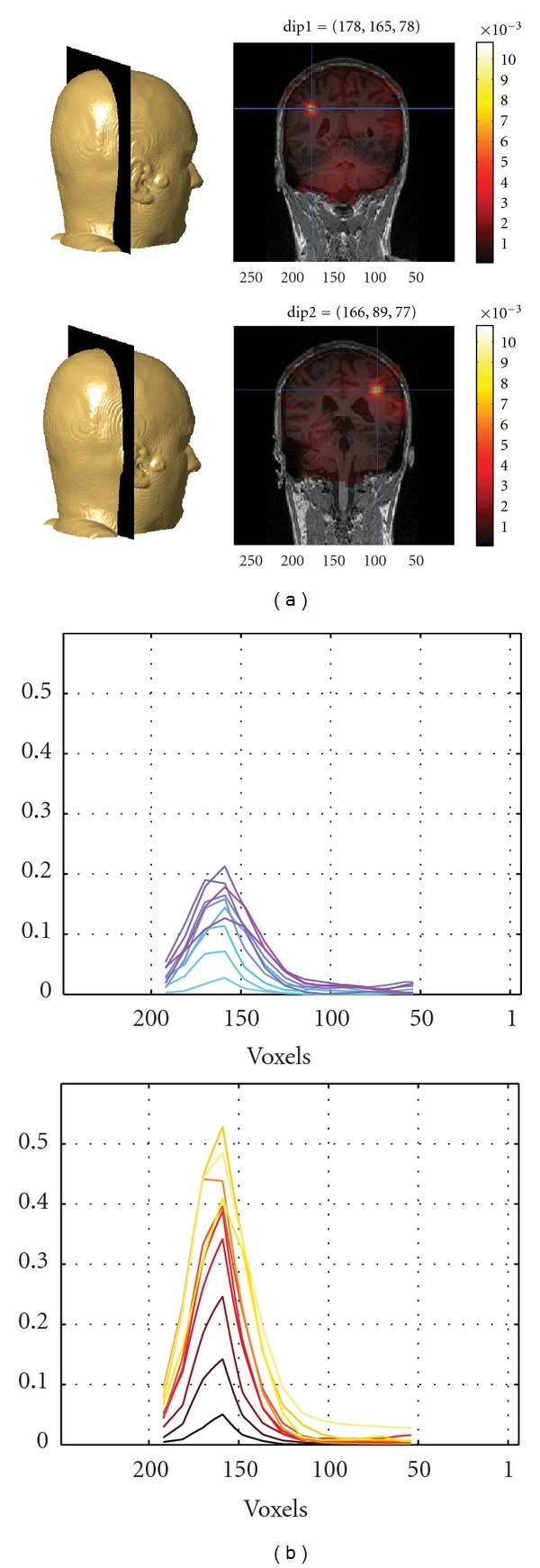
Results of the simulations. (a) The localization of two EMG-correlated sources is achieved in consecutive steps: with a standard DICS method (top-right panel, sagittal slice) and with DICS combined with regional suppression (bottom right panel). The leftmost source (dip1) is suppressed with a ratio of 30 (source coherence ratio), while the rightmost source is enhanced with a ratio of 17. (b) The localization of a single source with ten different SNR levels (cyan is the lowest and magenta is the highest) is achieved with DICS (top panel). The same simulated source+noise datasets are processed after local removal of noise by means of subspace reprojection (black is the lowest SNR, yellow is the highest).

Simulation 1 shows that the nulling beamformer is able to suppress the left source and enhance the right one (Figure 3). Note that the dataset contains the time courses of both sources, but the classic beamformer approach could localize only the source with the highest SNR (see Figure 1). Due to this cancellation effect, it is impossible to proceed in the localization of putative weaker sources without recurring to alternative techniques as the one described here.

Simulation 2 shows the increase in SNR (Figure 3(b)) after the application of the subspace reprojection using the selected ROI. The upper picture shows the coherence profiles along the coronal slice (depicted in Figure 3(a)) and each line represents the DICS result for a different dataset. The results with a low SNR are in light blue and the ones with a high SNR in magenta according to the color scheme convention named “cool” in MATLAB. Accordingly, the lower picture shows the results for the same datasets after the combination of subspace reprojection and DICS. The SNR color scheme increases from black to yellow (color map “hot” in MATLAB). Note the general increase of SNR of the “hot” curves with respect to the “cool” ones, caused by the noise reduction yielded by the aforementioned subspace reprojection technique.

### 3.2. The Subjects' Data

One of the five subjects (subject 5) did not show any muscular-MEG coherence at the sensor level and therefore was discarded from further analyses.

#### 3.2.1. The Results of Beamspace Reprojection

**Figure 4:**
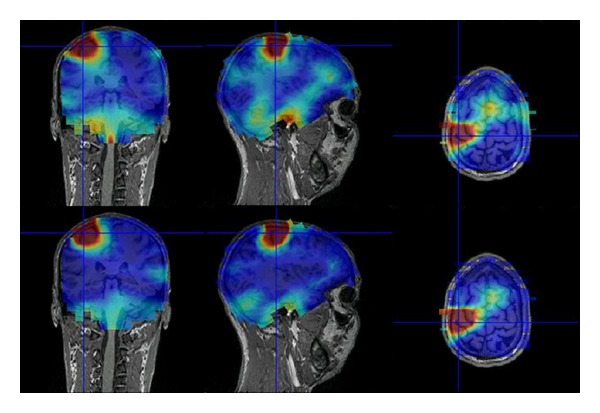
The subspace reprojection applied to subject 1. The three plots on top represent the coronal, sagittal, and transversal projections of the three-dimensional coherence map, before the application of beamspace reprojection. The three plots on the bottom depict the same projections of estimated activity, after the reduction of spatial noise. The technique is used to enhance the visualization of the coherence map. Both sets of plots refer to the same color scale (interval ranges from 0 to 0.008 of estimated coherence), and “jet” colormap is used (blue = 0 and red = 0.008). The plots are interpolated from a coarser grid (about 1 cm resolution along the Cartesian axes).

One representative subject is selected to show the results of the applied technique (subject 1).  Figure 4 shows the results of DICS beamformer before (upper row) and after (lower row) the application of the subspace reprojection technique on the preprocessed dataset. This illustrative example shows that the technique is effective in removing the noise from the spatial locations outside the region of interest of the coherence's peak.

#### 3.2.2. The Results of the Nulling Beamformer

**Figure 5:**
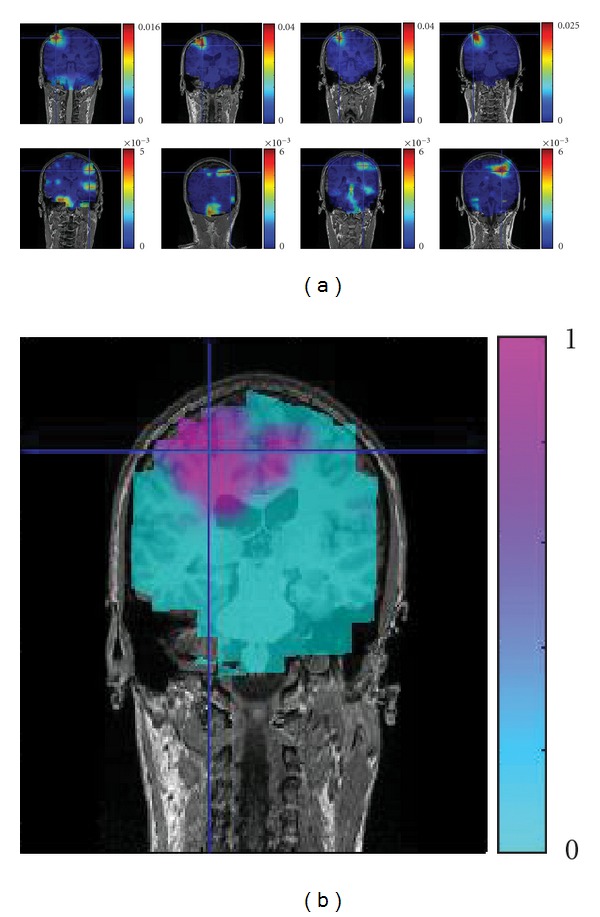
(a) The panel shows the results of DICS approach in a coronal slice. The resulting maps are used for the localization of the main source (upper row) and of the secondary source (lower row) in four of the five subjects. (b) The panel shows the map of the P* values (P* = 1 − *P*) for subject 2, as a result of a randomization test on the trials of the coherence (*N*_perm_ = 100). All plots are interpolated versions of a coarser grid (about 1 cm grid resolution along the Cartesian axes).

The nulling beamformer is effective in the suppression of the main CMC source as visible in Figure 5. The plots in panel (a) represent the coherence maps of all good subjects (from left—subject 1 to right—subject 4) in a coronal projection, before the suppression (top row) and after the suppression (lower row). The maps are thresholded by a minimum level of coherence, corresponding to a 5% statistical significance, as calculated by the randomization test previously described. It is noteworthy to mention that the newly calculated coherence threshold is smaller than the theoretical one as in (9) by a factor of 5 (e.g., theoretical = 0.01, randomized = 0.002).  Figure 5(b) represents the P* = (1 − *P*) value map for subject 2, as calculated from the randomization test. The *P* value map is used to calculate the threshold for the coherence maps (the minimum of coherence among all significant P*-map voxels constitutes the randomized threshold).

## 4. Conclusions and Discussion

The application of the proposed analysis pipeline for the localization of CMC sources has evidenced the presence of main CMC activations and putative secondary sources in simulations and real data. Despite the difficulties of beamformer approaches to correctly localize multiple phase-locked sources, (a phenomenon called source cancellation and demonstrated in formula (29) of VanVeen's paper, [16]), the application of the here proposed sequential procedure is able to reconstruct the underlying individual sources. In case of a strong primary and a weak secondary source, convincing results have been obtained. In case of similarly strong sources a modified procedure might be required.

The metric of coherence is computationally efficient for the localization of motor sources and can be equally applied to any protocol in which the external signal induces a phase-locked response in the brain. The rationale of applying the described pipeline is the need for a technique that identifies the presence (or absence) of more than one source correlated with EMG. The further step could be the use of these “seeds” for the identification of additional neural aggregates taking part in the motor processes.

Analyses on the single subject level are evidently able to localize the involved oscillatory sources. Although long recording times are needed to improve the SNR and therefore the estimation of the correct beamformer weights, the recording sessions could be divided in training and test sessions, where the first is used to localize the CMC sources and the second to track their time courses. Having a good estimate of the beamformer weights means being able to efficiently extract the activity of the underlying sources and therefore accomplish the ambitious task of a real-time connectivity analysis between EMG and MEG sources.

Based on the significant cortico-muscular coherence found in the present study we cannot unambiguously tell which brain region is the driving force for motor control. Muscular control could be due exerted through (1) both ipsi- and contralateral sources, (2) mainly a contra-lateral source that drives peripheral muscles directly from contralateral motor cortex and at the same time indirectly via the ipsilateral motor cortex, and (3) another location that drives both contra- and ipsilateral cortices and eventually the peripheral muscles. There is indication that the cerebellum could be involved in this network as well (see lower plots in Figure 5). On this aspect the results are inconclusive and only evidence a clear ipsi-lateral secondary source. According to this evidence the claims about a functional relation between the sources remain merely speculative. The application of methods like directed coherence or partial directed coherence might be capable to identify the causal flow of information in the network.

The physiological significance of our findings is that during an isometric contraction task multiple cortico-motor sources are recruited that constitute a putative network of motor coordination.

In conclusion, the application of advanced and time-efficient techniques for source suppression/enhancement has made possible the identification of cortico-muscular brain activations that otherwise would not be localizable with standard techniques.
